# Characteristics and Outcomes of Anti–Vascular Endothelial Growth Factor Therapy in a Large Cohort of Patients with Pachychoroid Neovasculopathy

**DOI:** 10.1177/24741264251381985

**Published:** 2025-10-31

**Authors:** Carolin Aizouki, Amit V. Mishra, Graeme K. Loh, Nishanthan Ramachandran, Mark D. J. Greve, David Ehmann, Parampal S. Grewal, Mark E. Seamone

**Affiliations:** 1 University of Alberta, Faculty of Medicine and Dentistry, Edmonton, Alberta, Canada; 2 Alberta Retina Consultants, Edmonton, Alberta, Canada; 3 University of Alberta, Department of Ophthalmology and Visual Sciences, Edmonton, Alberta, Canada

**Keywords:** anti-VEGF, cessation, choroidal neovascular membrane, pachychoroid, pachychoroid neovasculopathy, photodynamic therapy, treat and extend

## Abstract

**Purpose:** To examine the long-term visual and morphologic outcomes in a large series of patients with pachychoroid neovasculopathy (PNV) treated with intravitreal anti–vascular endothelial growth factor (anti-VEGF) injections. **Methods:** A retrospective, observational study of anti-VEGF injections in 249 eyes of 237 patients with PNV at 1 retina center over 7 years. **Results:** Mean patient age was 65.5 years and mean follow-up was 1.91 years (range 3 months to 7.14 years). At baseline, mean best-corrected visual acuity (BCVA) was 20/60 Snellen, with an improvement of 2.30 ETDRS letters by the study endpoint (*P* = .04). From baseline to endpoint, mean central subfield thickness decreased by 78.2 μm, and mean choroidal thickness decreased by 35.4 μm (each *P* < .05). Treat-and-extend was utilized in 192 eyes (77.9%), with a 42.3% recurrence rate on extension. Treatment cessation was trialed in 70 eyes (28.1%), of which 53 eyes required no further treatment. Adjunct photodynamic therapy was utilized in 34 eyes (13.7%), resulting in a mean vision improvement of 0.44 ETDRS letters, compared with a mean improvement of 2.30 ETDRS letters in patients who received anti-VEGF only (*P* = .6). **Conclusions:** Anti-VEGF therapy improved BCVA and anatomic features in this large cohort of patients with PNV. Continued treatment was required in 196 eyes (78.7%) at the study endpoint.

## Introduction

Pachychoroid neovasculopathy (PNV) is a vision-threatening disease within the spectrum of pachychoroid disorders.^1^ In addition to PNV, the pachychoroid disease spectrum includes central serous chorioretinopathy (CSCR), pachychoroid pigment epitheliopathy, peripapillary pachychoroid disease, and focal choroidal excavation.^1–3^ These conditions share the pachychoroid phenotype, which includes features of choroidal thickening, reduced choriocapillaris, dilated choroidal veins, retinal pigment epithelial (RPE) dysfunction, and choroidal neovascularization (CNV).^2–4^ PNV has the following differentiating characteristics: presence of pachychoroid, type 1 macular neovascularization (MNV), presence of pachyvessels near the MNV, and absence of active CSCR, age-related macular degeneration (AMD), and myopic degeneration.^5^ Diagnosing PNV can be challenging because of its resemblance to neovascular AMD (nAMD). However, substantial distinctions exist between PNV and nAMD.^6^ Compared to nAMD, PNV has increased choroidal thickness, minimal soft drusen, earlier age at onset, increased RPE irregularities, hyperpermeability of the choroidal vasculature, and distinct inflammatory cytokine patterns.^6,7^ Despite the differences between nAMD and PNV, treatment for PNV has been inferred from established nAMD protocols.

Intravitreal anti–vascular endothelial growth factor (anti-VEGF) injections are the standard treatment for nAMD.^8^ The MINERVA study was the first to describe the use of anti-VEGF injections for treatment of CNV outside of nAMD.^9^ Subsequent studies have evaluated the use of anti-VEGF therapy in PNV.^4,8–17^ Studies comparing the outcomes of intravitreal anti-VEGF for PNV versus nAMD have shown no significant difference in best-corrected visual acuity (BCVA) improvement following treatment.^4,12,13^ Other studies have compared PNV treatment efficacy between aflibercept and ranibizumab, with findings showing that aflibercept is superior to ranibizumab in achieving a dry macula.^11,15^ These current studies have drawbacks, such as the limited number of patients assessed, making it challenging to make inferences about treatment outcomes.

The purpose of the present study was to investigate the long-term visual and morphologic outcomes and treatment paradigms in a large cohort of more than 200 PNV patients treated with intravitreal anti-VEGF injections in a single center.

## Methods

### Study Design

The study was designed as a retrospective, observational study of patients with PNV whose eyes were treated with intravitreal anti-VEGF injections. Each patient provided written informed consent to participate. Medical records of 237 patients with PNV (249 eyes) who attended a single retina center, Alberta Retina Consultants, over a 7-year period (2016–2023) were reviewed. A retrospective search of the patients’ electronic medical records (HealthQuest database) was performed. Inclusion criteria included patients diagnosed as having PNV who were treated with anti-VEGF injections and followed up for longer than 3 months. Furthermore, PNV was diagnosed in patients who had pachychoroid features, type 1 MNV, absence of drusen or only pachydrusen, and absence of polypoidal lesions.^5,6^ Patients with CNV related to other etiologies and those with <3 months’ follow-up were excluded.

### Outcome Measures

Baseline demographic characteristics of the patients were recorded. All patients underwent ophthalmic examination, including BCVA measured using Snellen eye charts, optical coherence tomography (OCT), and slit-lamp biomicroscopy. In addition, patients underwent spectral-domain OCT (Heidelberg Spectralis). Enhanced-depth imaging was used to measure choroidal thickness.

Our primary outcome was the BCVA measured at baseline and throughout the study. OCT characteristics were used to evaluate central subfield thickness (CST), presence of pigment epithelial detachments (PEDs), pachydrusen, intraretinal fluid (IRF), and subretinal fluid (SRF), choroidal thickness, and subretinal hyperreflective material (SRHM) at baseline and at best-vision BCVA timepoint. CST was defined as the mean retinal thickness from the internal limiting membrane to Bruch’s membrane (BM) within a 1 mm diameter around the foveola.^8^ PEDs were measured as the distance between the RPE and BM at its highest point. The presence of pachydrusen was determined as a sub-RPE deposit larger than 125 μm in size. Choroidal thickness was measured as the distance between the BM and the sclerochoroidal junction at the foveal center. OCT analysis was performed by 3 vitreoretinal fellows.

Additional measures included the number of anti-VEGF injections administered, duration of treatment, PNV recurrence rate with treat-and-extend (T&E), and PNV recurrence rate with treatment cessation. Recurrence was defined as worsening IRF/SRF, as demonstrated on OCT imaging, requiring a decrease in the anti-VEGF treatment interval. The use of adjunct treatment using half-dose, half-fluence (half-half) photodynamic therapy (PDT) was analyzed, including visual and morphologic outcomes in the subgroup who were treated with PDT. The half-dose was 3 mg/m^2^ with half-fluence of 25 J/cm^2^.

### Statistical Analysis

Statistical analysis was performed using R statistical software (version 3.6.2.; R Foundation for Statistical Computing). The groups were normally distributed and *t*-test was utilized for comparing groups.

## Results

Baseline characteristics are presented in Table 1. A total of 249 eyes of 237 patients with PNV were included in the study (43% female). The mean age of patients at baseline was 65.5 years (range 26–95 years). Mean baseline BCVA was 61.0 ETDRS letters (range, 20/20–20/4000; Snellen equivalent, 20/60). Follow-up time was a mean 1.91 years (range, 3 months to 7.14 years). A total of 6.5% of patients with developing PNV followed up in our clinic had pachychoroid features. Previous CSCR was present in 17.0% of eyes (n = 42), with 64.3% of eyes (n = 27) having received previous treatment with PDT or focal laser therapy.

**Table 1.** Baseline Characteristics of Patients With PNV (249 Eyes, 237 Patients).

Characteristic	Value
Age, mean (range), years	65.5 (26–95)
BCVA, mean (range) Snellen	20/60 (20/20–20/4000)
Follow-up, mean (range)	1.91 years (3 months to 7.14 years)
History of CSCR, n (%)	42 (17)
Previous CSCR treatment, n (%)	27 (11)

Abbreviations: BCVA, best-corrected visual acuity; CSCR, central serous chorioretinopathy; PNV, pachychoroid neovasculopathy.

### Visual Outcomes

Table 2 shows a mean visual gain of +2.30 ETDRS letters by the end of the study (increasing from mean 61.0 to 63.3 ETDRS letters; *P* = .04). Peak BCVA gain occurred at a mean of 41 weeks after treatment initiation (gain of +11.6 ETDRS letters; *P* < .05), with a gradual regression toward baseline vision. Mean treatment interval at the best-vision BCVA timepoint was 5.9 weeks (range 3.6–19 weeks, SD 2.7 weeks).

**Table 2.** Visual and Morphologic Characteristics of Patients at Baseline and After Anti-VEGF Treatment (249 Eyes, 237 Patients).

Characteristic	Baseline	Posttreatment	Change from Baseline	*P* Value
BCVA (mean ETDRS letters)	61	63.3	+2.30	.04
Choroidal thickness (mean µm)	342	306	−36	<.05
Central subfield thickness (mean µm)	350	272	−78	<.05

Abbreviations: anti-VEGF, anti–vascular endothelial growth factor; BCVA, best-corrected visual acuity.

### Imaging Characteristics

As shown in Table 2, the mean choroidal thickness and CST at baseline was 342 μm and 350 μm, respectively. After treatment, mean choroidal thickness significantly decreased by 36 μm (decreasing from 342 μm to 306 μm; *P* < .05). No relationship was observed between choroidal thickness and number of anti-VEGF injections administered. The mean CST significantly decreased by 78 μm after treatment (*P* < .05).

At baseline, PEDs were the most common imaging finding (89.2% of eyes) followed by SRF (86.3% of eyes), pachydrusen (78.3% of eyes), IRF (69.9% of eyes), and SRHM (49.8% of eyes). In eyes with SRF on presentation, 31.7% had a complete resolution of fluid over the course of the study. A dry macula was achieved in 137 eyes (55.0%) that showed fluid (IRF/SRF) on imaging within the study period. Among patients who received adjunct PDT therapy, 14 eyes (40%) had a dry macula by study end.

### Treatment Paradigms

All study eyes received anti-VEGF treatment. The mean number of total injections was 13.9, with a mean of 7.28 injections per year. In patients still receiving treatment at the end of the study, the mean injection interval was 7.77 weeks. The majority of eyes were started on treatment with bevacizumab (70.9%), with fewer eyes started with aflibercept (28.6%) or ranibizumab (0.427%). A switch from bevacizumab/ranibizumab to aflibercept occurred in 16.2% of eyes. BCVA gain with bevacizumab was +2.93 ETDRS letters, compared to a gain of +3.13 ETDRS letters with aflibercept (*P* = .94). In patients who had a switch of agent during the study, there was a BCVA gain of +0.135 ETDRS letters by study end, which was significantly smaller than that seen in the other 2 treatment groups (each *P* < .05).

There was a statistically significant difference in macula drying at study end between the aflibercept and bevacizumab groups. A dry macula was achieved in 69 of 112 eyes treated with aflibercept and 56 of 137 eyes treated with bevacizumab (*P* < .05). No statistically significant differences between the aflibercept and bevacizumab groups were seen in the CST (mean 95.9 μm vs 76.3 μm; *P* = .18) and the choroidal thickness (mean 40.0 μm vs 35.9 μm; *P* = .20) at study end.

**Figure 1.**
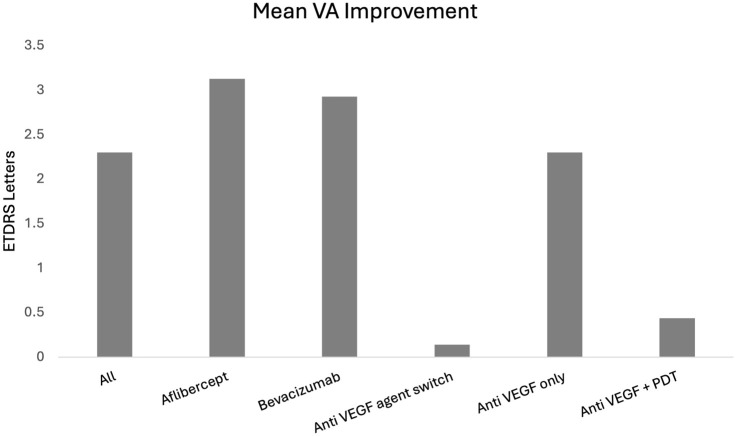
Mean BCVA improvement across different treatment groups reported in ETDRS letters.

Adjunct treatment with half-half PDT was utilized in 34 eyes (13.7%) during the study period. The mean BCVA gain by study end in the group receiving PDT and anti-VEGF was 0.44 ETDRS letters, compared to a gain of 2.30 ETDRS letters in the anti-VEGF only group. The difference in BCVA improvement was not statistically significant between these 2 groups (*P* = .6), as shown in Figure 1.

### Recurrence Rates in Treat-and-Extend Group

T&E was utilized in 192 eyes (77.9%) during the study. There was a mean recurrence rate of 42.3% (81 eyes) on extension, at a mean interval of 7 weeks. Visual gain in patients without recurrence on extension (111 eyes) was a mean increase of +3.79 ETDRS letters, compared to a mean increase of +1.04 ETDRS letters in those who experienced recurrence on extension (Table 3). This difference was not statistically significant (*P* = .33). When comparing patients who had no recurrence on T&E and those who had recurrence, there was no statistically significant difference in baseline age (mean 64.4 years vs 67.7 years; *P* = .08), baseline CST (mean 349.1 μm vs 366.6 μm; *P* = .2), or baseline choroidal thickness (mean 340.0 μm vs 349.2 μm; *P* = .5). There was a significant difference in the number of anti-VEGF treatments required, with patients who experienced recurrence on extension requiring more injections than those with no recurrence (mean 21.0 vs 12.6 injections; *P* < .05).

**Table 3.** BCVA Changes With Anti-VEGF Treatment in Patients With or Without PNV Recurrence After T&E and Treatment Cessation.

Follow-up Protocol	Posttreatment BCVA^a^	Posttreatment BCVA Gain^a^	*P* Value vs Recurrence Group
T&E^b^			
No recurrence on extension	64.8	+3.79	.33
Recurrence on extension	62.0	+1.04	
Treatment Cessation^c^			
No recurrence on cessation	64.4	+3.40	.98
Recurrence on cessation	64.5	+3.53	

Abbreviations: anti-VEGF, anti–vascular endothelial growth factor; BCVA, best-corrected visual acuity; PNV, pachychoroid neovasculopathy; T&E, treat-and-extend.

a Values are mean ETDRS letters.

b n = 192 eyes (111 eyes without recurrence, 81 eyes with recurrence).

c n = 70 eyes (53 eyes without recurrence, 17 eyes with recurrence).

### Recurrence Rates in Treatment Cessation Group

Cessation of treatment was trialed in 70 eyes (28.1%), with 53 of these eyes (75.7%) requiring no further treatment. The mean BCVA gain in eyes with no IRF/SRF recurrence on treatment cessation was +3.40 ETDRS letters, compared to a mean BCVA gain of +3.53 ETDRS letters in those who had recurrence on cessation and required further treatment (17 eyes) (Table 3). The difference between these groups was not statistically significant (*P* = .98). Of the eyes in which recurrence occurred after treatment cessation, 41.1% no longer required treatment by the end of the study. Of those who experienced recurrence after cessation and still required treatment, the mean injection interval was 8.7 weeks. When comparing those with and those without recurrence on cessation, there was no statistically significant difference in baseline CST (mean 340.5 μm vs 361.1 μm; *P* = .5), baseline choroidal thickness (mean 371.2 μm vs 344.9 μm; *P* = .3), or baseline number of injections (mean 9.0 vs 11.6; *P* = .2). There was a significant difference in patient age, with the population experiencing recurrence after treatment cessation being younger (mean age 55.5 years) than the group who did not experience recurrence after cessation (mean age 65.4 years; *P* < .05).

## Discussion

This study examined the long-term visual and morphologic outcomes and treatment paradigms in 249 eyes of 237 patients with PNV treated with intravitreal anti-VEGF injections. Our results showed statistically significant visual gains, peaking at a mean of 41 weeks and a treatment interval of 5.9 weeks, followed by a regression back to baseline vision. T&E was utilized in 77.9% of eyes, with a recurrence rate of 42.3% on extension. Treatment cessation was trialed in 28.1% of eyes, with a 75.7% success rate. There was a statistically significant reduction in choroidal thickness and CST from baseline to study end, by a mean of 36 μm and 78 μm, respectively. Of the eyes presenting with SRF, 31.7% had complete resolution of fluid by the end of the study period. In all eyes that had IRF/SRF, 55% had no fluid in the macula by the end of the study. There was no statistically significant difference in BCVA gain between the bevacizumab and aflibercept groups; however, those receiving aflibercept had a statistically significant higher rate of macular drying compared to those receiving bevacizumab. Adjunct treatment with PDT did not result in a statistically significant BCVA gain compared to the anti-VEGF only group.

Previous studies have examined the efficacy of T&E in PNV treatment. These studies had a loading phase where patients received 3 monthly anti-VEGF injections.^8,12^ The treatment interval was increased or reduced by 2 weeks depending on the presence of exudative changes, up to a maximum of 12 weeks. Matsumoto et al compared T&E in 42 PNV eyes versus 60 nAMD eyes.^12^ They found no statistically significant difference in BCVA gains and central macular thickness (CMT) reduction between the 2 groups at 2 years. In a study by Montero Hernández et al, 31 PNV eyes were initially treated with aflibercept followed by T&E. They found no statistically significant improvement in BCVA but did observe a significant reduction in CMT over 2 years.^8^ Our study demonstrated statistically significant improvements in BCVA and reduction in choroidal thickness and CST with anti-VEGF treatment. We describe PNV recurrence on extension to be characterized as worsening of IRF/SRF accumulation, resulting in regression of the treatment interval, which was observed in 42.3% of patients for whom T&E was utilized. This is a novel finding, as preceding studies did not indicate their rate of recurrence on extension.

Padrón-Pérez et al investigated the impacts of anti-VEGF treatment in PNV on choroidal thickness and on BCVA in 18 eyes of Caucasian patients.^10^ They observed a statistically significant reduction in choroidal thickness following anti-VEGF treatment, which correlated with the number of injections given. Furthermore, they saw no statistically significant BCVA improvement over the 12-month study period. Schworm et al investigated anti-VEGF treatment in 14 PNV eyes, finding no significant improvement in BCVA at 2 years.^18^ They found a reduction in choroidal thickness, which was correlated with the number of injections administered. The results of preceding studies align with our findings with regard to significant reductions in choroidal thickness and CST. In our patient cohort, no relationship was identified between the number of intravitreal anti-VEGF injections and choroidal thickness reduction. Contrary to previous studies, which assessed no more than 31 eyes,^8,10,18^ we believe our study to be adequately powered to show a statistically significant improvement in BCVA with treatment over 2 years. In fact, Padrón-Pérez et al were able to show a statistically significant improvement in BCVA when they removed from their study 2 eyes that had shown a vision loss of more than 20 EDTRS letters and that were perceived to be outliers.^10^ Furthermore, our study had a mean peak BCVA gain of 11.6 ETDRS letters at 41 weeks after initiation of anti-VEGF treatment. There was subsequent regression back to baseline vision, with mean BCVA gain by the end of the study being +2.30 ETDRS letters. Should this trend continue, further follow-up of our cohort may reveal insignificant BCVA gains.

Statistically significant BCVA gains were described in several studies. Hikichi et al treated 50 PNV eyes with anti-VEGF therapy.^19^ Aflibercept and ranibizumab were administered to 68% and 32% of eyes, respectively. They observed statistically significant improvement in BCVA over the 1-year study period, along with complete resolution of SRF in 86% of eyes. Jung et al reported improved BCVA with anti-VEGF treatments in 54 PNV eyes, with no significant difference between patients treated with aflibercept and those treated with ranibizumab.^15^ They observed complete resolution of SRF in 82.6% of eyes in the aflibercept treatment group and 51.6% of eyes in the ranibizumab group. Lastly, Elfandi et al compared the efficacy of aflibercept treatment between 27 PNV eyes and 63 nAMD eyes.^4^ A significant BCVA improvement occurred over the study period, with 77% of the eyes with PNV achieving a dry macula. The results from these studies concur with our findings of significant improvement in BCVA following anti-VEGF treatment. However, it is of note that the relative rate of complete resolution of SRF and dry macula was lower in our study, at 31.7% and 55.0%, respectively. One possible explanation is the choice of anti-VEGF agent. Our study had 70.9% of patients treated with bevacizumab, 28.6% with aflibercept, and 0.427% with ranibizumab. Conversely, the preceding studies only used aflibercept and ranibizumab.^4,15,19^ Karasu et al distinguished treatment efficacy between bevacizumab, aflibercept, and ranibizumab.^17^ They found that CMT reduction was not significant in those treated with bevacizumab but was significant in those treated with aflibercept or ranibizumab, and BCVA improvement was not significantly different across the 3 treatment groups. In our study, the rate of eye drying was found to be statistically significantly different between the aflibercept and bevacizumab groups (*P* < .05); treatment with aflibercept and treatment with bevacizumab achieved a dry macula in 69 of 112 eyes and 56 of 137 eyes, respectively. Furthermore, we observed a greater reduction in CST (mean 95.9 μm vs 76.3 μm; *P* = .18) and choroidal thickness (mean 73.9 μm vs 54.4 μm; *P* = .25) with aflibercept compared to bevacizumab; however, this difference was not statistically significant. This may explain our relatively lower rate of SRF resolution compared to other studies, as the majority of our patients were treated with bevacizumab.

The etiology underlying type 1 neovascularization in PNV is thought to differ from nAMD. Upregulation of VEGF in PNV may be attributed to focal RPE disturbances and inner choroid diminution overlying pachyvessels.^2,19^ Angiogenesis may also be triggered by chronic inflammation of the choriocapillaris.^20^ Furthermore, choroidal thickening in PNV may be attributed to increased choroidal hydrostatic pressure, which may not be targeted by anti-VEGF; instead, anti-VEGF therapy may work to attenuate leakage of fluid into the stroma by pachyvessels.^18,19^ PDT induces choroidal hypoperfusion, helping reduce choroidal hydrostatic pressure and congestion. A study by Lee and Lee demonstrated resolution of SRF with PDT treatment in 28 PNV eyes refractory to anti-VEGF monotherapy.^21^ Jung et al utilized PDT in 6 treatment-resistant eyes and achieved a dry macula by 3 months.^15^ Hikichi et al compared the use of anti-VEGF and half-dose PDT in a total of 88 eyes, finding that both treatments successfully controlled SRF and had similar improvements in BCVA.^19^ Adjunct PDT treatment was utilized in 13.7% of eyes in our study. In agreement with the findings from the Hikichi et al study, the BCVA gains in patients treated with adjunct PDT was not significantly different compared with the gains in the anti-VEGF only group. In our patient population treated with PDT rescue therapy, 40% had a dry macula at study end, while SRF was completely resolved in 31.7% of all study eyes. It is possible that patients with persistent SRF may benefit from adjunct PDT. Given that our patients received PDT as rescue therapy, the minimal BCVA gains in our study may be a reflection of sample selection of cases resistant to treatment. Although PDT has benefits in resolving SRF, it carries the risk of damaging normal choroidal vasculature and RPE.^15^ Taking this into account, it appears that PDT should be reserved for use as a rescue therapy in anti-VEGF–resistant PNV.

Limited studies have examined cessation of anti-VEGF treatment in patients with PNV. One of the existing studies, by Kinoshita et al, explored outcomes with anti-VEGF cessation in 101 treatment-naive eyes with nAMD or PNV.^22^ At the end of their study period, 26.7% of eyes had successful treatment cessation, compared to 21.3% of eyes with successful treatment cessation in our study. Kinoshita et al found that successful cessation was associated with absence of disease activity at 12 weeks and with fewer recurrences throughout the study period. Furthermore, they reported that patients’ baseline phenotypic characteristics do not predict successful treatment cessation. Our study found that baseline CST and baseline choroidal thickness were not significantly different between those who did and those who did not experience IRF/SRF recurrence with treatment cessation. Kinoshita et al’s study combined findings from both nAMD patients and PNV patients when describing treatment cessation.^22^ To the best of our knowledge, our study is the first to describe anti-VEGF treatment cessation in PNV alone.

Our study has several limitations. It is a retrospective, single-center study that was nonrandomized and included 27 eyes with previous PDT treatment for CSCR. Selection bias is always possible in a retrospective study. Given that the catchment area for our clinic is large, there is a possibility of loss to follow-up that may skew the results. This study’s strengths include the large number of cases and long-term follow-up at a single center. Our data are derived from consecutive cases of PNV over a 7-year period, with collection of significant patient data such as BCVA and OCT imaging findings at every visit (including injection visits).

Our study describes the efficacy of anti-VEGF treatments in PNV. Statistically significant improvements in visual acuity and reduction in choroidal thickness were seen with treatment. Further investigations into understanding prognostic factors that may contribute to the success of the T&E protocol and cessation of treatment for patients are warranted.
